# Active phytochemicals of *Pueraria tuberosa* for DPP-IV inhibition: in silico and experimental approach

**DOI:** 10.1186/s40200-017-0328-0

**Published:** 2017-11-21

**Authors:** Shivani Srivastava, Priya Shree, Yamini Bhusan Tripathi

**Affiliations:** Department of Medicinal Chemistry, Institute of Medical Sciences, Banaras Hindu University, Varanasi, U.P India

**Keywords:** In silico, Postprandial, Incretins, DPP-iv, Oral glucose tolerance test, Fasting, In vivo

## Abstract

**Background:**

We had earlier reported that the extract of *Pueraria tuberosa* significantly inhibits DPP-IV enzyme, resulting in glucose tolerance response in rats. In this study, we have explored the active phytochemicals responsible for this potential. The results have been validated in both fasting and postprandial states in the plasma of normal rats and also in fasting blood and intestinal homogenates of diabetic models.

**Methods:**

*Pueraria tuberosa* water extract (PTWE) was administered to normal Charles Foster rats for 35 days and to diabetic model (65 mg/kg bw) for 10 days. After treatments, oral glucose tolerance test (OGTT) and insulin was done for 90 min, and the changes in the levels of GLP-1, GIP, and DPP-IV activities were monitored in fasting and postprandial states. In the case of the diabetic model, DPP-IV activity was measured in intestinal homogenate and basal insulin in plasma. The components of PTWE were analyzed via HPLC-MS based on their chemical formula, molecular mass, and retention time. Using the molecular docking study, we have selected the top five components having strong binding energy with DPP-IV.

**Results:**

The increase in secretion of GLP-1 and GIP was significantly higher in the postprandial state when compared to fasting condition. GLP-1 plasma concentration increased by 5.8 and 2.9 folds and GIP increased by 8.7 and 2.4 folds in PTWE and control rats, respectively. In contrast, the postprandial decrease in DPP-IV specific activities was recorded at 2.3 and 1.4 folds. The response in OGTT and insulin was also consistent with these changes. In comparison to diabetic controls, PTWE-administered rats showed decreased DPP-IV activity in the intestine, leading to enhanced basal insulin concentration. Through molecular docking, we found Puerarone and Robinin to be the most potential phytochemicals of PTWE for DPP-IV inhibition. Binding energy (kcal/mol) and dissociation constant (pM) of Robinin with DPP-IV protein were found to be 7.543 and 2,957,383.75, respectively. For Puerarone, it was 7.376 and 3,920,309, respectively.

**Conclusions:**

Thus, this study provides the novel active components that contribute to the DPP-IV inhibitory property of PTWE.

## Background

In the past few decades, incretin therapy has become one of the most widely used approaches for treating diabetes. It includes two types of medicines, i.e., dipeptidyl peptidase IV (DPP-IV) inhibitors (vildagliptin, sitagliptin, alogliptin, linagliptin and saxagliptin) [1] and glucagon-like peptide-1 (GLP-1) receptor agonists (liraglutide, exenatide) [2]. Glucose-dependent insulinotropic polypeptide (GIP) and GLP-1 are responsible for the homeostatic regulation of glucose-induced insulin secretion. Their half-life in plasma is less than 2 min. due to their rapid inactivation by ubiquitously found proteolytic enzyme DPP-IV (CD 26). The serine protease DPP-IV cleaves the dipeptides from amino terminus, which includes alanine or proline residue in position 2, thus making the two hormones, GIP and GLP-1, biologically inactive [3]. Hence, synthetic preparation or identification of potential natural DPP-IV inhibitors is a crucial target for the treatment of diabetic patients.

With respect to DPP-IV inhibition, several plants like *Allophylus cominia* (AC) [4], *Berberis aristata* (BA) [5], and *Mangifera indica* (MI) [6] have been considered. Active phytochemicals, such as cirsimaritin, hispidulin, naringenin, resveratrol, luteolin, apigenin, flavone, and berberine [7–9], of these plants have been screened through in vivo and in silico parameters. The ancient Ayurvedic approach is considered as the most effective and safest treatment method if taken with proper knowledge of dose and preparations [10]. In this regard, many plants against diseases like cardiovascular problems such as hypertension and hyperlipidemia as well as with anti-diabetic potential (e.g., *Trigonella foenum-graecum* [11], *Rhuscoriaria* L. [12], *Melissa officinalis* [13], *Berberis aristata, Tamarindus indica* [14]) have been discovered; another such plant is *Pueraria tuberosa* (PT), which reportedly shows positive effects in the treatment of diabetes [15, 16], nephropathy [17, 18], and inflammation [19]. One of its constituents, i.e., puerarin, has been extensively studied for stress [20, 21], hyperglycemia [22], and incretin pathways [23] in diabetes. However, there is limited information on active phytochemical constituents of PT water extracts (PTWE) with respect to DPP-IV inhibition. In our previous work, we have reported the inhibitory effect of PTWE on DPP-IV activity [15]*.* In this paper, we have extended our study using the same model of non-diabetic rats for chronic in vivo treatment up to 35 days under fasting and glucose-fed conditions. We correlated the results with the levels of GLP-1, GIP, OGTT and insulin. We also checked the DPP-IV inhibition potential in STZ-induced chronic diabetic model. In addition, we also screened the major phytochemicals for direct DPP-IV inhibitory property through in silico molecular docking.

## Method

### Sample preparation

PT roots were purchased from the Ayurvedic Pharmacy, Banaras Hindu University. 30 g powder of PT roots was extracted with 8 volumes of distilled water. When the volume was reduced to its one-fourth, it was filtered with cloth. The total yield obtained by this process was 30% [15].

### Animal design

The protocol was approved by the Institute Ethical Committee, Institute of Medical Sciences, Banaras Hindu University, India. For normal rats study, 12 male Charles Foster rats of the same age group (3–4 weeks) were divided into two groups (6 each): group 1 for PTWE and group 2 for control. Group 1was administered PTWE at a dose of 50 mg/100 g bw for 35 days. For diabetic model, 12 male Charles Foster rats were administered STZ (65 mg/kg bw) injection. After 60 days, the diabetic rats (glucose level above 200 mg/dL) were divided into two groups: group 3 as a diabetic control and group 4 for PTWE treatment (Diabetic PTWE). After treatment, the rats were sacrificed along with normal rats (Normal Control) in order to isolate intestine (duodenum). The inner parts of the intestine were washed and homogenized with 1× PBS to prepare 20% homogenate.

### Chemicals and materials used

DPP-IV fluorometric assay kit, GLP-1 & GIP Enzyme Immunoassay (EIA) Kit and Gly-pro-p-nitroanilide (GPPN) were purchased from Sigma Aldrich.

### Glucose tolerance test

The rats were administered anesthesia, and their basal blood samples were collected in EDTA-containing tubes. Then they were orally administered PTWE at the dose of 50 mg/100 g bw. After 2 h of PTWE treatment, all the rats were given glucose orally at the dose of 2.5 g/kg bw. Then blood was again collected through tail at 45 min and 90 min following glucose loading. Glucose concentration in blood was determined using glucose strips (S.D check) at every step of blood collection.

### Insulin

Insulin was measured through Immulite 1000 (Siemens) solid phase, two-site chemiluminescent immunometric assay.

### GLP-1 and GIP concentration in plasma

GLP-1 and GIP concentrations in blood plasma were measured by their EIA Kit.

### DPP-IV specific activity in plasma

DPP-IV activity was done by adding 95 μl GPPN (0.2 mM) as substrate in the mixture of 65 μlTrisHCl (50 mM, 7.5 pH) and 10 μl plasma. Absorbance was taken immediately and after 20 min at 405 nm. The protein concentration was measured via the Bradford method.

### DPP-IV activity in homogenate

DPP-IV activity in homogenates was measured by their respective fluorometric assay kit (Sigma MAK088).

### Molecular docking

For molecular docking study, the 3D protein structures of DPP-IV (PDB ID-4FFV) were retrieved from the RCSB Protein Data Bank (http://www.rcsb.org). The 2D structures of precious phytochemicals (Robinin, Puererone, Anhydrotuberosin, Daidzin, Tuberosin, Puerarin 4′,6′-diacetate, Tuberostan, Puetuberosanol, Puerarostan, 3-O-methylanhydrotuberosin, Puererin, 4-methoxypuerarin, Stigmasterol, Anthocyanins, Genistin, Hydroxytuberosone, Tectoridin, β-sitosterol, Biochanin A, Biochanin B, Quercetin, Genistein, and Daidzein) from PTWE were retrieved from PubChem compound database (http://pubchem.ncbi.nlm.nih.gov/) and converted into 3D structure using Discovery Studio 3.0 [24]. The protein receptor model was prepared by optimizing protein model geometry and removing ligands and other heteroatoms using the Discovery Studio 3.0. Further, the protein models were taken for active site prediction using Discovery Studio 3.0 and MetaPocket (http://projects.biotec.tu-dresden.de/metapocket/) [25] and subjected to docking studies. Molecular docking calculation was done using the YASARA software [26]. For this, 25 selected phytochemicals from PT were taken for docking with DPP-IV. In YASARA, the receptor and ligand files were used to set target and play macro. The macro file dockrun_mcr was used to calculate the interaction between the receptor and the selected ligands individually. There were 25 runs of all the ligands and receptor files for docking calculation. Then the object files (docked complex) were visualized using the YASARA software and converted to PDB files for 2D-3D interaction using Discovery studio 3.0. The result log files obtained from YASARA were used for docking calculation. The docking results were sorted by binding energy [kcal/mol] and dissociation constant [pM]. The compounds with more positive binding energy showed a stronger interaction and inhibition activity with receptors.

### Statistical analysis

Statistical analysis was determined by one-way ANOVA following post hoc test using Dunnett’s and Tuckey test by IBM SPSS Statistics Software.

## Results

### Antidiabetic parameters

#### DPP-IV inhibition

**Fig. 1:**
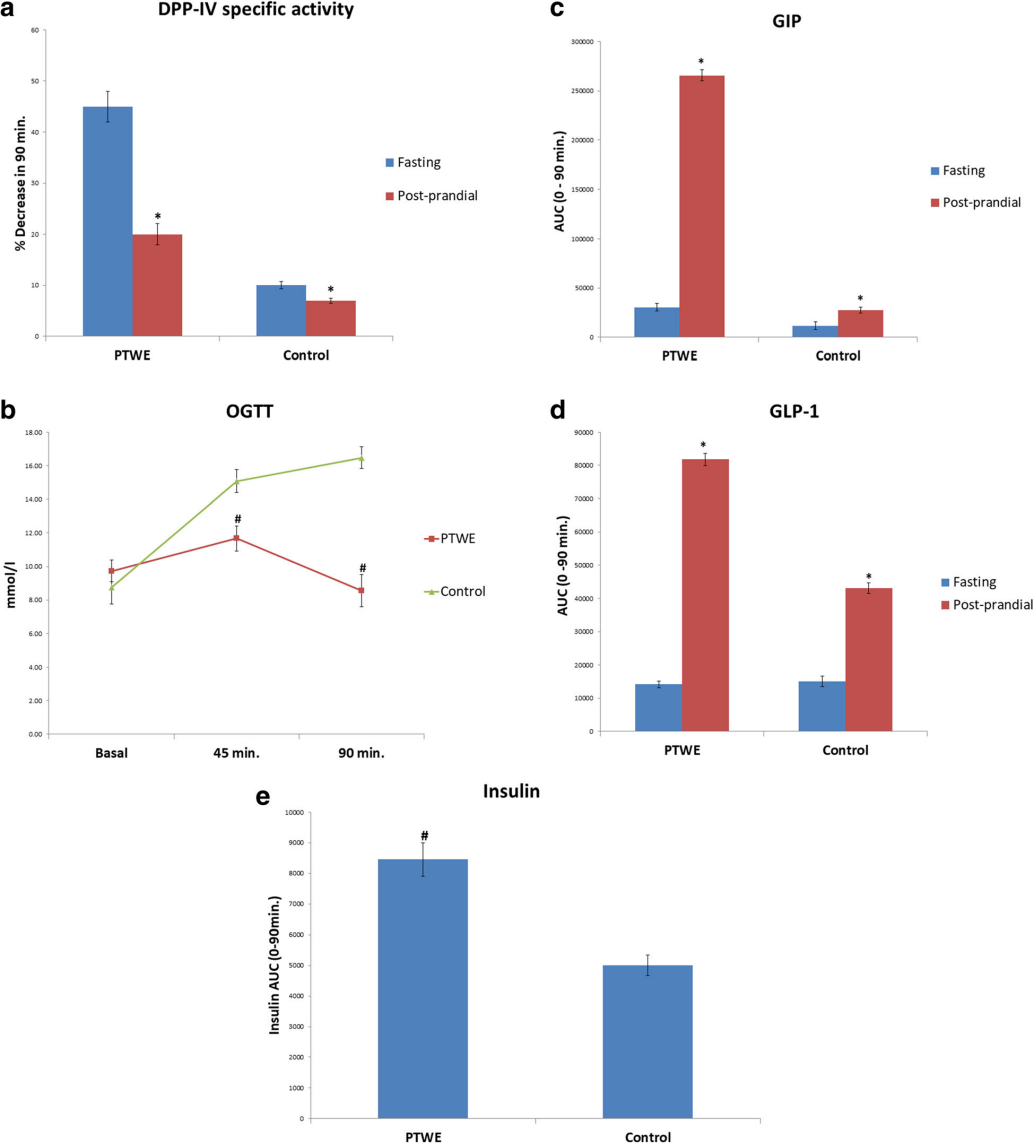
Comparision of fasting and postprandial effects of PTWE and vehicle in normal rats’ plasma; (**a**) Percent decrease of DPP-IV specific activity from fasting to post-prandial state after 90 min. of glucose load, (**b**) Oral glucose tolerance test up to 90 min, (**c**) glucose-dependent insulinotropic polypeptide (GIP), (**d**) glucagon-like peptide-1 (GLP-1) and (**e**) Insulin. Values represented as mean ± SD (*n* = 6 in each group). *p < 0.05 compared with fasting group.# *p* < 0.05 compared with control group

As shown in Fig. 1, PTWE means PTWE-treated normal rats and Control means Vehicle-treated normal rats. At fasting, the plasma DPP-IV specific activity was higher compared to the postprandial state. After 90 min of glucose administration, a decrease in plasma DPP-IV specific activity was observed at 1.4 folds lower than the fasting stage. Interestingly, in PTWE-treated rats, this postprandial plasma DPP-IV level was 2.3 folds lower than the fasting plasma DPP-IV value (Fig. 1 (a)). This suggests that glucose administration reduces the plasma DPP-IV level, but in the presence of PTWE, this reduction was more significant, indicating higher GLP-1 and GIP values in plasma and better hypoglycemic state in these rats.

**Fig. 2:**
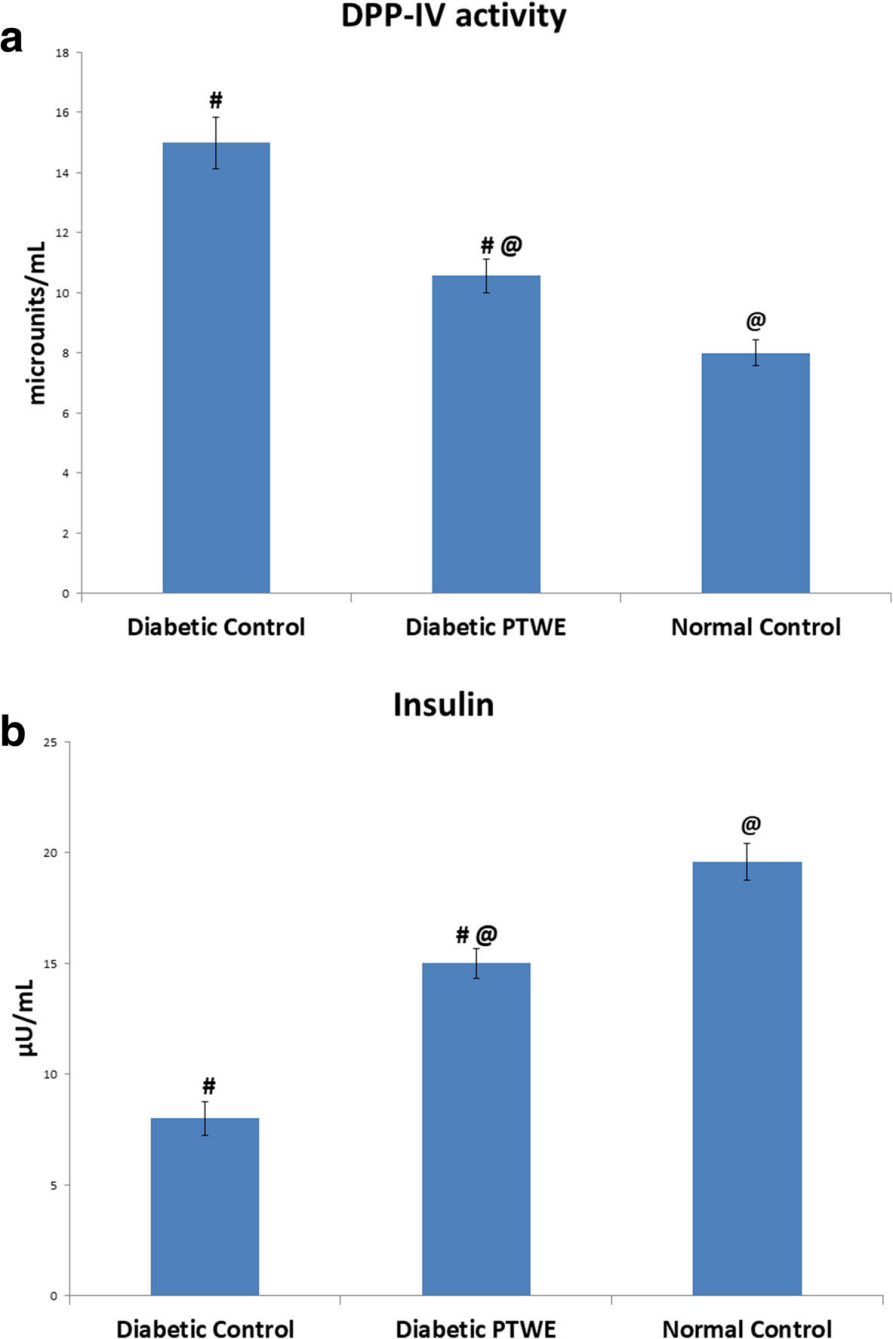
Effect of PTWE in diabetic model of rats; (**a**) DPP-IV activity in intestinal homogenate and (**b**) Plasma insulin. Values represented as mean ± SD (*n* = 6 in each group). # *p* < 0.05 compared with normal control group. @*p* < 0.05 compared with diabetic control group

In the case of diabetic model, the oral administration of PTWE (50 mg/100 g bw) for 10 days significantly reduced the STZ-mediated stress-induced DPP-IV activity in intestinal homogenates (Fig. 2 (a)). It was observed that after 60 days of STZ injection, the increase in intestinal DPP-IV activity was approximately 1.9 fold as compared to normal control, but in diabetic PTWE group, this increase was 1.3 fold. Thus, the cocktail of PTWE also acts as a potential inhibitor of DPP-IV activity in the case of chronic diabetes.

### Oral glucose tolerance test

PTWE treatment also enhanced the glucose tolerance capacity measured at 45 and 90 min (Fig. 1 (b)). This suggests that a normal individual after PTWE treatment consumes more carbohydrates, without challenging the threshold of insulin release from the pancreas. In other words, pretreatment with PTWE may reduce the load on pancreas while consuming high carbohydrate diet. Thus, PTWE can be used as a prophylactic measure to prevent the onset of hyperglycemia-mediated stress on the pancreas.

### Plasma incretin secretion

GLP 1 and GIP in plasma were enhanced after glucose loading. After 90 min of glucose loading, the secretion of GLP-1 was found to be increased by 5.8 folds in the case of PTWE-treated rats and 2.9 folds in control rats. Also for GIP levels, a similar trend was observed with 8.7 and 2.4 folds increase in PTWE-treated rats and control rats, respectively (Fig. 1 (c), (d)).

### Insulin

Area under curve for insulin between basal up to 90 min in PTWE rats was found to be 1.7 folds higher than that in control rats (Fig. 1(e)).

In comparison to diabetic controls, PTWE significantly increased (1.9 fold) the basal insulin concentration in plasma (Fig. 2 (b)).

### Molecular docking

**Fig. 3:**
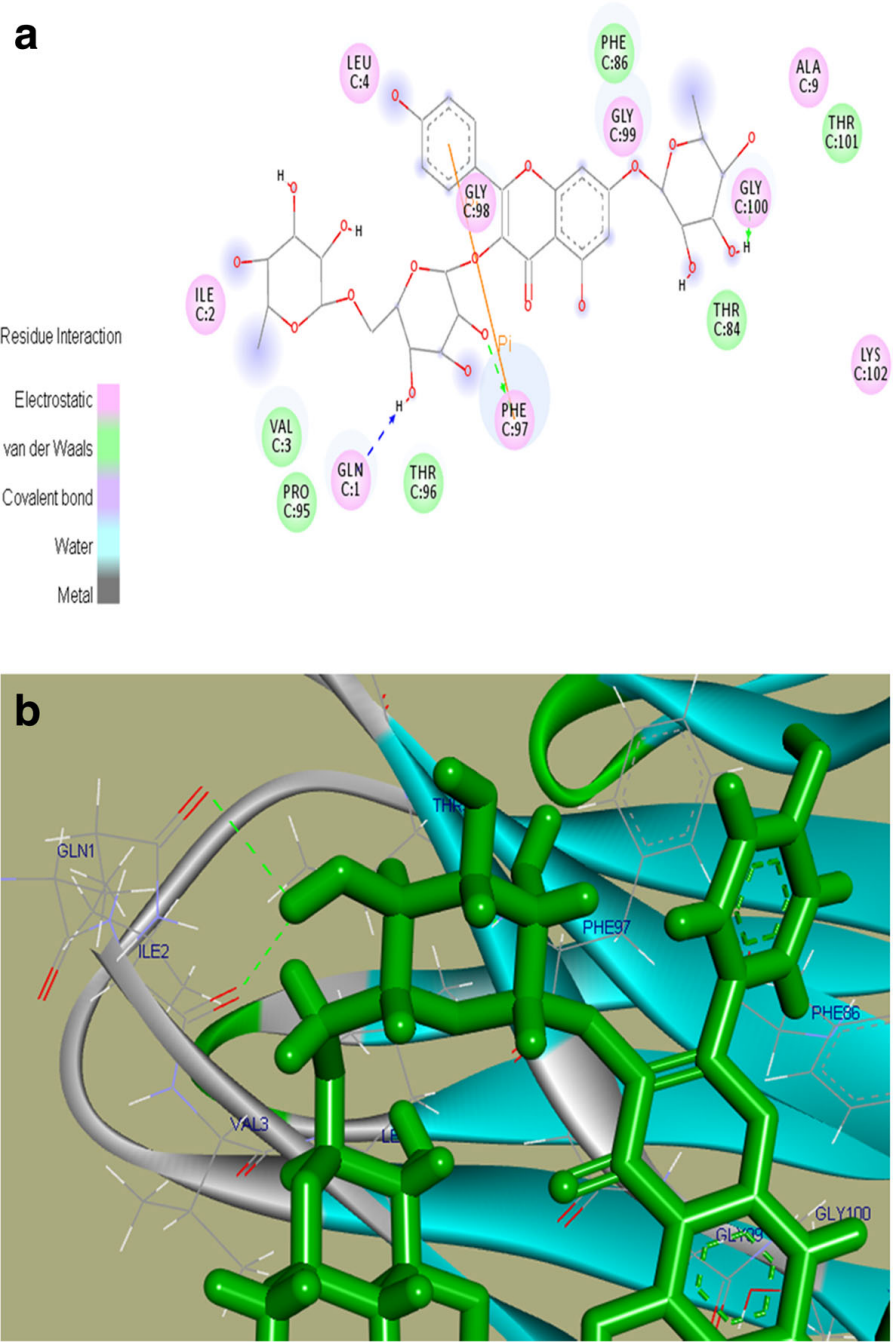
**a**. 2D structure of Robinin with DPP-IV protein. The residues in light pink color show electrostatic interaction while the green color residues show van der Waals interaction. The residues GlnC^1^, PheC^97^, and GlyC^100^ show direct interaction and hydrogen bonding with Robinin, and the residue PheC^97^shows Pi-Pi interaction with Robinin; 3(**b**) 3D structure of Robinin with DPP-IV protein

**Fig. 4:**
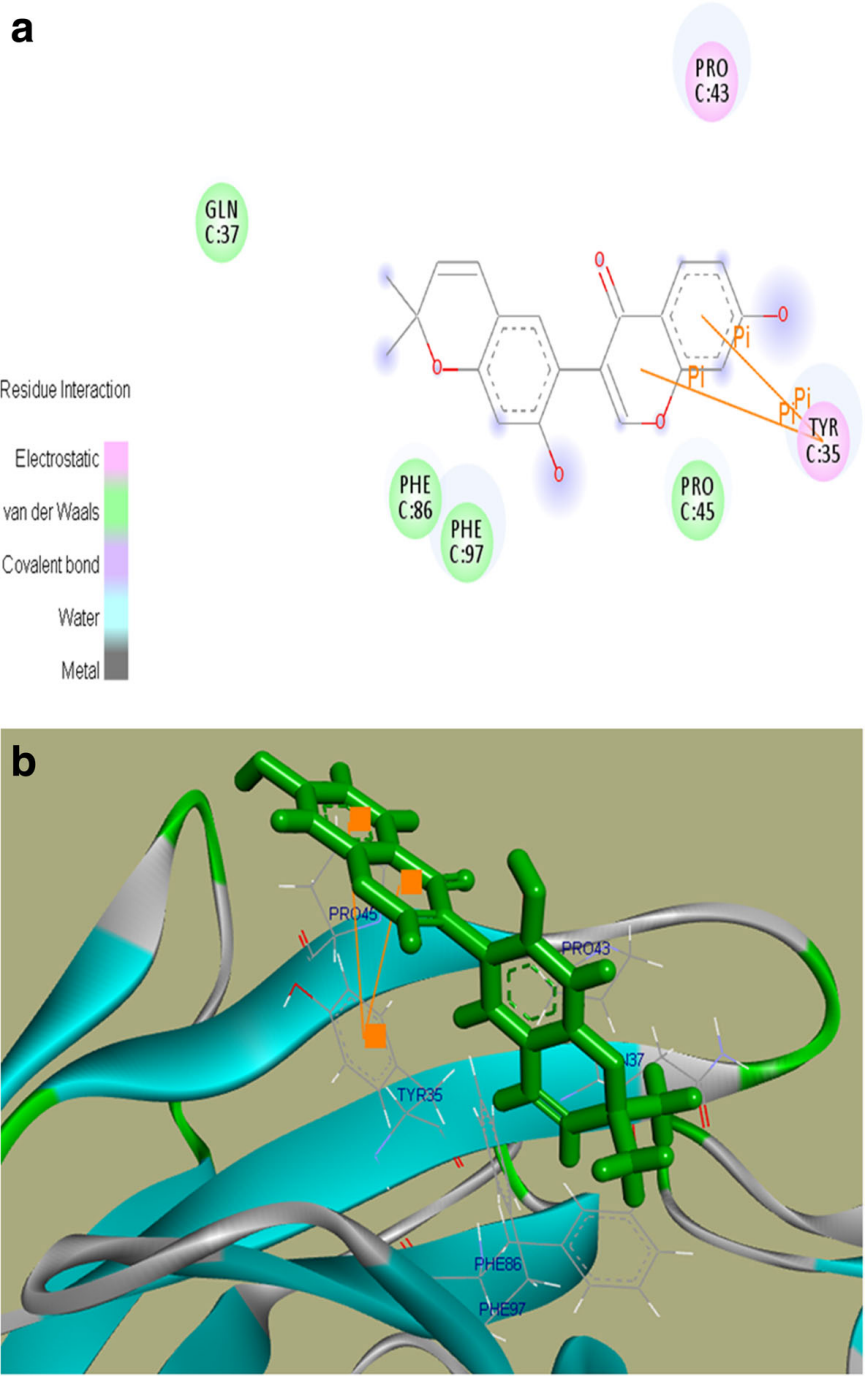
**a**. 2D structure of Puererone with DPP-IV protein. The residues in light pink color show electrostatic interaction while the green color residues show van der Waals interaction. The residue TyrC^35^ shows Pi-Pi interaction with Puererone; 4(**b**) 3D structure of Puererone with DPP-IV protein

We found that out of the 25 selected phytochemicals from PTWE, only two compounds, namely Robinin and Puererone, showed best interactions with DPP-IV protein. They had binding energy of 7.543 kcal/mol and 7.376 kcal/mol and dissociation constant of 2,957,383.75 pM and 3,920,309 pM, respectively (Table 1). Further, Robinin and Puererone were taken for interactive visualization using Discovery studio 3.0. It was found that the active-site amino acid residues GlnC^1^, IleC^2^, ValC^3^, LeuC^4^, AlaC^9^, ThrC^84^, PheC^86^, ProC^95^, ThrC^96^, PheC^97^, GlyC^98^, GlyC^99^, GlyC^100^, ThrC^101^, and LysC^102^of DPP-IV were involved in the interactions with Robinin. The residues GlnC^1^, PheC^97^, and GlyC^100^ showed direct interaction and hydrogen bonding with Robinin, suggesting inhibition activity. The Pi-Pi interaction was formed by residue PheC^97^ (Fig. 3(a), (b)). For Puererone, the active-site amino acid residues TyrC^35^, GlnC^37^, ProC^43^, ProC^45^, PheC^86^, and PheC^97^ of DPP-IV were involved in the interactions. The residue TyrC^35^ showed Pi-Pi interaction with Puererone (Fig. 4(a), (b)). The positive binding energies of the two phytochemicals with DPP-IV protein showed that they have strong binding affinity and inhibition activity towards DPP-IV protein.

**Table 1:** Binding energy and Dissociation constant of Robinin and Puererone with DPP IV protein

Compound name	Binding energy (kcal/mol)	Dissociation constant (pM)
Robinin	7.543	2,957,383.75
Puererone	7.376	3,920,309.00
Anhydrotuberosin	7.149	5,750,604.5
Daidzin	7.042	6,888,831.5
Tuberosin	6.965	7,844,898.00

## Discussions

Through the molecular docking of PTWE phytochemicals obtained by HPLC–MS (complete data not shown) with DPP-IV, we found five potential DPP-IV inhibitors, i.e., Robinin, Puererone, Anhydrotuberosin, Daidzin, and Tuberosin (Tables 1, 2). Of them, Robinin and Puererone showed more positive binding energies with DPP-IV (Figs. 3, 4). This indicates that the two novel compounds act as DPP-IV inhibitors and could play a vital role in the treatment of diabetes.

**Table 2:** Chemical profiling of potential phytochemicals of PTWE obtained by HPLCMS

Compound name	Chemical formula	Molecular mass (g/mol)	Retention time (minute)
Robinin	C33 H40 O19	740.664	3.198
Puererone	C20 H16 O5	336.343	0.667
Anhydrotuberosin	C20 H16 O4	320.344	0.525
Daidzin	C21 H20 O9	416.382	8.32
Tuberosin	C20 H18 O5	338.359	0.897

It has been previously studied that plasma DPP-IV activity increases at fasting [27, 28]. Fasting DPP-IV activity is more enhanced in type 2 diabetic patients than in normal patients due to uncontrolled hyperglycemia or enhanced CD26 T cell activity response, but the process is not well understood [29]. Overall fasting must create a stress response in the body that must be compensated through diet intake. Glucose, the most crucial element present in many diets, is known to be responsible for the secretion of GLP-1 and GIP according to the needs of the healthy body. Thus, after glucose loading, the body has a self-adaptive approach to compensate the excess glucose present in the body by secreting GLP-1 from L cells and GIP from K cells of the intestine. The GLP-1 and GIP bind to their respective receptors, i.e., GLP-1R and GIP–R, leading to the activation of insulin signaling pathway [3]. In our study, PTWE intensified these glucose regulatory homeostatic properties through DPP-IV inhibition response by 1.64 times (Fig. 1 (a)). PTWE also enhanced GLP-1 plasma secretion by 2 times (Fig. 1 (d)) and plasma GIP by 3.7 times (Fig. 1 (c)), leading to increased insulin secretion and thus, increased glucose tolerance capacity in the body (Fig. 1 (b) & (e)). Here, PTWE with glucose reduces the stress response provided by fasting more effectively than glucose alone. On the other hand, PTWE also upregulates plasma insulin concentration and inhibits DPP-IV activity in the intestine of diabetic rats (Fig. 2). Therefore, this herbal drug provides a hypoglycemic response through DPP-IV inhibition pathway. Flavonoids play a role in DPP-IV inhibition [8, 4, 30] and have also been studied as GLP-1 receptor agonists [31]. In our previous study, we have proved PTWE as a competitive inhibitor of DPP-IV [15]. Through computational modeling study, flavones were found to be competitive inhibitors and dock directly into all three active sites of DPP-IV [8]. Thus, the positive binding energy of DPP-IV with active isoflavones Robinin and Puerarone present in PTWE must be the most probable reason this plant tuber acts as a DPP-IV inhibitor. According to an in silico study, Puerarone shows strong affinity to VEGFR 1 and VEGFR 2 along with 93.881% human intestinal absorption [32]. This effect of PT on VEGF has been studied in our lab but in kidney nephropathy [33]. DPP-IV inhibition has been found to activate CREB and improves signaling pathway of islet vascularization through VEGF-A/VEGFR 2 [34]. So, it could be concluded that the constituent Puerarone of PTWE works on angiogenesis through the above pathways involving DPP-IV inhibition and vascular endothelial growth factors. Of the 2000 known flavonoids, Robinin is one of the most common dietary compounds. In the gut, Robinin has been found to be hydrolyzed to kaempferol by fecal flora [35]. According to another independent study, kaempferol has the potential to inhibit DPP-IV activity [36]. The above studies, thus, suggest that the gut flora plays an important role in DPP-IV inhibition mechanism. It means in the diabetic model, the activity of beneficial microbes gets reduced. Here, we can hypothesize that, in addition to Robinin, PTWE contains some other dietary compounds responsible for enhancing these activities of gut microbes. It has been studied that DPP-IV inhibitor bis-pyranoprenyl isolated from ethyl acetate fraction from aerial parts of *Polygala molluginifolia* contributes to GLP-1 secretion through the stimulation of calcium influx in the intestine. The overall mechanism involves voltage-dependent calcium channels, phospholipase C, protein kinase C, and stored calcium [30]. As the compositions of all DPP-IV inhibitory plants found in nature are different, the above-discussed pathway must be the common mechanism, with minor modifications for each plant, through which GLP-1 secretion could be enhanced through DPP-IV inhibition. The minor modifications in the mechanism are based on the phytochemicals found specifically in the plants. So, active phytochemicals like Robinin and Puerarone found in PTWE should be studied individually for their DPP-IV inhibition mechanism. The markedly available DPP-IV inhibitors have many adverse effects such as upper respiratory tract infection, nasopharyngitis, headache, urinary tract infection, and so on [37]. PT tubers possess many components such as daidzin, puerarin, puerarone, genistein, puetuberosanol, tuberostan, tuberosin, and puerarin 4′,6′-diacetate [32]. Thus, the use of PTWE could be more beneficial due to the synergistic response provided by all these phytomolecules and its multi-targeted action on many inflammation and stress-linked diseases discussed above. Thus, PTWE provides the cheapest and safe treatment for diabetes in comparison to synthetic drugs.

## Conclusions

PTWE enhances the glucose homeostatic potential of body through DPP-IV inhibitory pathway via its active components Robinin and Puerarone. Thus, PTWE and its novel components must be considered as an anti-diabetic drug with respect to DPP-IV inhibition.

## Abbreviations

DPP-IV: Dipeptidyl Peptidase IV
GIP: Glucose-Dependent Insulinotropic Polypeptide
GLP-1: Glucagon Like Peptide-1
OGTT: Oral Glucose Tolerance Test
PTWE: *Pueraria tuberosa* water extract
